# Impact of Indoor Radon Exposure on Lung Cancer Incidence in Slovenia

**DOI:** 10.3390/cancers16081445

**Published:** 2024-04-09

**Authors:** Mojca Birk, Tina Žagar, Sonja Tomšič, Katarina Lokar, Ana Mihor, Nika Bric, Miran Mlakar, Vesna Zadnik

**Affiliations:** Epidemiology and Cancer Registry, Institute of Oncology Ljubljana, 1000 Ljubljana, Slovenia; tzagar@onko-i.si (T.Ž.); stomsic@onko-i.si (S.T.); klokar@onko-i.si (K.L.); amihor@onko-i.si (A.M.); nbric@onko-i.si (N.B.); mmlakar@onko-i.si (M.M.); vzadnik@onko-i.si (V.Z.)

**Keywords:** indoor radon exposure, lung cancer, Bayesian hierarchical model, population attributable fraction

## Abstract

**Simple Summary:**

Lung cancer is one of the most frequently diagnosed cancers worldwide. Radon is a radioactive gas whose concentrations can accumulate indoors. The long-term exposure to radon is considered carcinogenic to humans and is an important risk factor for lung cancer. Our epidemiological study investigated the impact of indoor radon exposure on the incidence of lung cancer in Slovenia over a period of 40 years. Around 60 newly diagnosed cases of lung cancer per year (out of a population of around 2 million) can be attributed to radon exposure in residential environments in the period 1978–2017, which corresponds to 5.5% of all lung cancer cases. The most important information that needs to be communicated to the Slovenian public and decision-makers about the health risk and about support for preventive measures is that living in areas with elevated radon levels is associated with a higher risk of lung cancer.

**Abstract:**

Indoor radon is an important risk factor for lung cancer, as 3–14% of lung cancer cases can be attributed to radon. The aim of our study was to estimate the impact of indoor radon exposure on lung cancer incidence over the last 40 years in Slovenia. We analyzed the distribution of lung cancer incidence across 212 municipalities and 6032 settlements in Slovenia. The standardized incidence ratios were smoothed with the Besag–York–Mollie model and fitted with the integrated nested Laplace approximation. A categorical explanatory variable, the risk of indoor radon exposure with low, moderate and high risk values, was added to the models. We also calculated the population attributable fraction. Between 2.8% and 6.5% of the lung cancer cases in Slovenia were attributable to indoor radon exposure, with values varying by time period. The relative risk of developing lung cancer was significantly higher among the residents of areas with a moderate and high risk of radon exposure. Indoor radon exposure is an important risk factor for lung cancer in Slovenia in areas with high natural radon radiation (especially in the southern and south-eastern parts of the country).

## 1. Introduction

Lung cancer is the most commonly diagnosed cancer and the leading cause of cancer-related deaths in men worldwide. In women, lung cancer is the second type of cancer both in newly diagnosed cancers and as the cause of cancer-related deaths [1]. In Slovenia, a Central European country with a population of around 2 million, lung cancer is the third most commonly diagnosed cancer. In 2020, 1554 new cases of lung cancer were diagnosed, which corresponds to 12.5% of all cancer cases in both sexes combined [2]. Smoking is the most important risk factor for lung cancer in the general population [3], and it is estimated that 85–90% of lung cancer cases are attributable to smoking [4].

Radon is a radioactive gas without smell, color, or taste, which is produced by the radioactive decay of uranium and radium in the earth’s crust. Uranium and its decay product radium are found in soil, rock, water, and building materials. The radon concentration in the outdoor air is low and not harmful to health, but the radon concentration can increase indoors [5]. The main source of indoor radon is the infiltration of radon from the soil through cracks and structural gaps in buildings [6]. The concentration of radon and its decay products depends on the structure of the earth’s crust, the soil parameters, and the weather conditions; indoors, it also depends on the construction and ventilation of buildings [7]—all of which influence the risk of lung cancer.

Radon and its decay products are deposited in the respiratory tract and emit ionizing radiation, which directly and indirectly damages the DNA in the epithelium of the respiratory tract and can lead to lung diseases and lung cancer [6,8]. The relation between the dose of radon radiation and the health effects appears to be linear and has no threshold value [9]. The International Agency for Research on Cancer has evaluated the evidence for the carcinogenicity of radon and classified radon as carcinogenic to humans in 1988 [10]. It is estimated that 3–14% of lung cancer cases can be attributed to radon [3]. To date, not many studies have been conducted on the geographical distribution of lung cancer incidence in Slovenia. In the 1980s, an epidemiological study was conducted using data from the Slovenian Cancer Registry to investigate the relation between the incidence of lung cancer and the Žirovski vrh uranium mine. Between 1981 and 1985, an increased incidence was found in men and women in settlements near the uranium mine, but a causal relationship could not be confirmed (it could not be determined whether this was due to exposure to radon, tobacco smoke, or chance) [11,12].

The Jožef Stefan Institute carried out the first radon measurements in Slovenia in 1969, at the Žirovski vrh uranium mine [13]. The Slovenian radon program was launched in 1990, and, since then, measurements have been carried out in kindergartens, schools, homes, and workplaces [13,14]. Since 2010, radon measurements have also been carried out in the soil near buildings with high indoor radon concentrations [15]. The results of the measurements of radon concentration and soil properties were used to create the Slovenian radon map [15,16], in which settlements and municipalities were categorized into areas of a low, moderate, and high risk of indoor radon exposure, with a threshold value of 200 Bq/m^3^ for a moderate risk and 300 Bq/m^3^ for a high risk [15]. High radon concentrations are mainly found in the Karst region in the south and south-east of Slovenia. Out of the 6032 settlements, the risk of indoor radon exposure is high in 1235 (21%) and moderate in 611 (10%) settlements. Out of 212 Slovenian municipalities, the risk of indoor radon exposure is high in 24 (11%) and moderate in 27 (13%) municipalities [15].

As the high risk of radon exposure has been demonstrated in some parts of Slovenia, the aim of our study was to investigate the association between indoor radon exposure and lung cancer incidence at the national level over a 40-year period and to find out how many lung cancer cases per year are attributable to radon exposure.

## 2. Materials and Methods

In our geographical descriptive correlational epidemiological study, four consecutive 10-year periods from 1978 to 2017 were analyzed. Two levels of geographical division were applied. To reduce heterogeneity, the smallest possible geographical areas were selected for the principal analysis—6032 settlements in Slovenia according to the 2017 definition. As several settlements have been renamed, changed their boundaries, or split into two new settlements over time, all older addresses had to be correctly reclassified into the settlements valid in 2017. Analyses by settlement were only possible for the 30-year period 1988–2017. In order to include the data for the period 1978–1987, for which only municipality-level data was available, all analyses were also repeated at the municipality level.

Three datasets were linked in the study at the level of the settlements: the risk of indoor radon exposure, the lung cancer incidence, and the background population. The information on radon exposure was provided by the Slovenian Radiation Protection Administration. As reported by Vaupotič and Gregorič [15], the indoor radon spatial activity concentration was measured in residential and working environments and averaged over the same lithological units (areas with the same physical properties of the rock) and settlements with more than two measurement points. The average radon concentration in the lithological units and settlements was divided into five radon concentration categories. The first category contained the units/settlements with an average radon concentration below 100 Bq/m^3^, the second those with a concentration between 100–200 Bq/m^3^, the third 200–300 Bq/m^3^, the fourth 300–600 Bq/m^3^, and the fifth category contained units/settlements with an average radon concentration above 600 Bq/m^3^. The polygons of the settlements were divided into a 50 m × 50 m grid, and each cell was assigned a lithological unit with a corresponding category. The average category across all the cells in the settlement was calculated. In this way, the average category of the settlement was calculated based on the lithological properties of the settlement. If there were two or more measurement points for the radon concentration in the settlement, the radon concentration category was determined based on the category of an average radon concentration in the settlements. The final radon concentration category of the settlement was the average of the average category based on the lithological properties and the category based on the average radon concentration in the settlement. Based on the categories, the settlements were categorized into three groups for the risk of indoor radon exposure—low (category of an average radon concentration 1 to 3), moderate (category 3 to 3.5), and high (category 3.5 to 5). The polygons of the municipalities were also divided into a 50 m × 50 m grid, where each cell was assigned the corresponding average category of radon concentration in the respective settlement, and the average category was calculated over all cells of the municipality. The average category was used to categorize the municipalities into three groups for indoor radon exposure risk, which were used for the analysis.

From the Slovenian Cancer Registry, we obtained data on lung cancer cases defined as C33–C34 according to the tenth revision of the International Classification of Diseases. The data on lung cancer cases were reported by sex, 5-year age groups, and place of residence at the time of diagnosis. The data on the population size in the settlements by sex and 5-year age groups were obtained from the Slovenian Statistical Office. We used data from the 1981, 1991, and 2002 censuses. For the period 2008–2017, data was available for each calendar year.

The number of new cancer cases depends heavily on the size and age structure of the population, which varies over time and space. To overcome this variation, the indirect method of standardization was applied, and the standardized incidence ratios (SIRs) were calculated by dividing the observed and expected cases [17]. A SIR of 1 means that the incidence in the geographical area being studied is the same as expected based on the incidence in the reference population, i.e., the Slovenian population in the same calendar period. A SIR above 1 is interpreted as the relative risk of developing lung cancer. The Slovenian national age-specific incidence rates were used to calculate the expected number of lung cancer cases. Analyses based on small geographical areas could lead to unreliable observations, as cancer is a rare disease and there are only a few or even zero cases in several units. To smooth the observed values and to account for spatial correlation and sampling variability, we used one of the Bayesian hierarchical spatial models, the Besag–York–Mollie (BYM) model fitted by the integrated nested Laplace approximation (INLA). The model proposed by Besage et al. [18,19,20] is as follows:

(1) $$\ln\frac{O_{i}}{E_{i}}=\ln E_{i}+a+H_{i\;}+S_{i},$$

where $O_{i}$ and $E_{i}$ represent the observed and expected number of the lung cancer cases in the *i*-th geographic area and a is the baseline relative risk of lung cancer in the entire analyzed area. The model also contains two types of random effects. $H_{i}$ represents the geographically independent heterogeneous component and has a normal distribution with mean zero and precision $\tau_{h}$. $S_{i}$ represents the spatial autocorrelation component. We evaluated the clustering of the smoothed SIRs across the Slovenian municipalities and settlements based on the ratio between the precision of the spatial autocorrelation component ($\tau_{s}$) and the precision of the heterogeneous component ($\tau_{h}$). If the $\tau_{s}/\tau_{h}$ ratio is less than 1, it indicates that the spatial autocorrelation component is more important than the heterogeneous component, as the variability of the spatial autocorrelation component is smaller than the variability of the heterogeneous component.

We included the risk of indoor radon exposure as an explanatory variable in the BYM model and thus excluded the effect of radon exposure in the smoothed SIRs. We calculated the proportion of cancer cases attributable to indoor radon exposure, the so-called Population Attributable Fraction (*PAF*) [21,22], as follows:

(2) $$PAF=1-\frac{1}{\sum_{1}^{3}p_{i}*{SIR}_{i}},$$

where *p_i_* is the proportion of the population in the *i*-th category of risk of indoor radon exposure and *SIR_i_* is the smoothed relative risk of lung cancer in each of the three *i*- categories of risk of indoor radon exposure. 

We combined the areas with a moderate and high risk of indoor radon exposure and analyzed the relative risk of lung cancer in the areas with a low and moderate/high risk of indoor radon exposure. We calculated the SIRs and interpreted the results as the relative risk of lung cancer. 

The analysis was conducted using the cancer incidence mapping tool CanMapTool (version 1.1) [20] and Rstudio (version 4.0.2; Posit Software, PBC, Boston, MA, USA) with the R package dplyr (version 1.0.2) for the data preparation. The shape files of the municipalities and settlements were provided by the Slovenian Surveying and Mapping Authority.

## 3. Results

### 3.1. The Burden of Lung Cancer in Slovenia

In the 40-year period 1978–2017, 8.1% of the Slovenian population lived in settlements with a high risk of indoor radon exposure and 7.3% with a moderate risk, making 15.4% of the population in total living in areas with a moderate or high risk of indoor radon exposure (Table 1).

In the 40-year period 1978–2017, there were 41,255 cases of lung cancer in Slovenia (Table 1). The place of residence at the time of the diagnosis was known for all lung cancer patients in the 40-year period analyzed, but the quality of this information is better for the period 1998–2017 than for earlier years.

**Figure 1:**
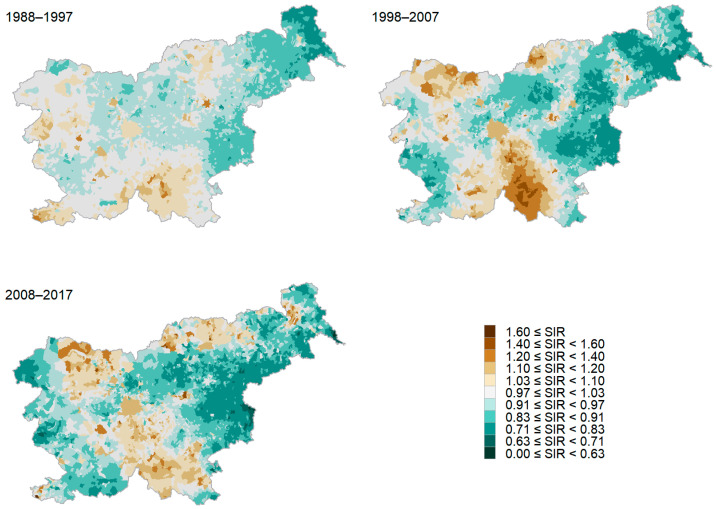
Smoothed standardized incidence ratio (SIR) of lung cancer by settlement in three consecutive 10-year periods.

The relative risk of developing lung cancer was higher for the residents of the coastal (in the south-west), the south-east, the central, and the north-west regions of Slovenia, as well as on the northern border from Koroška to Maribor and on the eastern border around Murska Sobota. In the period 2008–2017, the relative risk of lung cancer was statistically significantly higher than the Slovenian average for the residents of eleven settlements and six municipalities (Figure 1). The values of the ratio $\tau_{s}/\tau_{h}$ were below 1 for all four 10-year periods analyzed (between 0.0004 and 0.43 for the analysis by settlement and between 0.001 and 0.026 for the analysis by municipality), indicating that the random spatial component has a greater influence on the smoothed SIR distribution than the random heterogeneous component, i.e., maps of the smoothed SIRs of lung cancer across settlements (Figure 1) and municipalities show a geographical clustering.

### 3.2. The Burden of Lung Cancer in Slovenia and Indoor Radon Exposure

**Figure 2:**
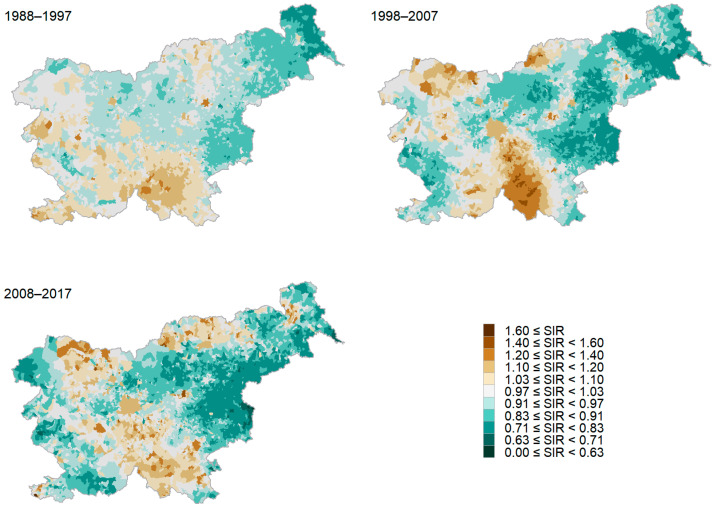
Smoothed standardized incidence ratio (SIR) of lung cancer by settlement in three consecutive 10-year periods. The effect of radon exposure is excluded.

In the BYM models, we included the risk of indoor radon exposure as an explanatory variable. The smoothed SIRs remained geographically clustered, as the value of the $\tau_{s}/\tau_{h}$ ratio in the analyses at the level of settlements and municipalities was less than 1 (between 0.001 and 0.06) in all four 10-year periods analyzed. The relative risk remained higher for the residents in the northern, central, and south-eastern parts of the country, even when we excluded the effect of radon exposure (Figure 2), confirming that radon exposure is not the main risk factor for lung cancer.

Depending on the time period, between 1.2% and 5.6% of the lung cancer cases in Slovenia could be attributed to exposure to radon in the residential environment when analyzed by settlement and between 2.8% and 6.5% when analyzed by municipality (Table 2). According to municipality-level analysis, 2278 lung cancer cases in Slovenia during the entire 40-year period were attributable to indoor radon exposure, which accounted for 5.5% of all lung cancer cases in Slovenia during this period.

The relative risk of developing lung cancer was higher among the residents of the areas with a moderate or high risk of indoor radiation exposure in all 10-year periods analyzed from 1978 to 2017 (Table 3). More specifically, the relative risk was 5% (95% confidence interval (CI): 0–11%) higher in the period 1978–1987, 7% (95% CI: 2–12%) higher in the periods 1988–1997 and 1998–2007, and 5% (95% CI: 0–9%) higher in the period 2008–2017.

In men living in the areas with a moderate or high risk of indoor radon exposure, the relative risk was statistically significantly higher in all periods analyzed. The relative risk was 7% (95% CI: 1–13%) higher in the period 1978–1987, 9% (95% CI: 3–15%) higher in the period 1988–1997, 8% (95% CI: 3–14%) higher in the period 1998–2007, and 5% (95% CI: 0–13%) higher in the period 2008–2017. Among women, the relative risk of developing lung cancer was not statistically significantly increased in areas with a moderate or high risk of indoor radon exposure in the 10-year periods 1978–1987, 1988–1997, and 1998–2007, but an increased relative risk of developing lung cancer could not be excluded, as shown by the upper limits of the 95% CI, which were above one.

## 4. Discussion

The aim of our study was to assess to what extent indoor radon exposure influences lung cancer incidence. The results showed that in Slovenia, for all four analyzed 10-year periods between 1978 and 2017, the map of the smoothed SIR of lung cancer is geographically clustered across municipalities and settlements and remains similarly distributed even when we had excluded the effects of indoor radon exposure in the models. The risk of lung cancer is higher in the northern, central, south-eastern, and coastal parts of the country.

Although indoor radon exposure is not the main risk factor in the general population, it is a significant health risk for people who live, work, or go to school in regions with a high risk of indoor radon exposure [3,10]. According to our analysis by municipality, almost 2300 lung cancer cases in Slovenia in the 40-year period 1978–2017 could be attributed to living in an area with a moderate or high risk of indoor radon exposure, which corresponds to 5.5% of lung cancer cases. In the most recent 10-year period 2008–2017, 4.3% of the lung cancer cases in Slovenia can be attributed to living in an area with a moderate or high risk of indoor radon exposure, as a more detailed analysis by settlement shows, which corresponds to about 570 cases. Our PAF results are in line with the WHO estimates of 3–14% of lung cancer cases being attributable to indoor radon exposure [3]. The percentage varies depending on geological properties and how governments and people respond to the increased risk of indoor radon exposure. For example, it is estimated that 8% and 5% of the lung cancer cases in Switzerland and Germany [23], as well as 4.7% of the lung cancer cases in 2010 in the UK, are attributable to radon exposure. In the UK, it is additionally estimated that 6% of lung cancer deaths per year are due to indoor radon exposure [24].

In order to reduce the health risks to individuals and populations, the World Health Organization has recommended a national reference level for radon concentrations in residential buildings of 100 Bq/m^3^, which must not exceed 300 Bq/m^3^ [25]. Researchers are not entirely in agreement about the threshold level of radon concentration in the air at which the risk of radon-induced lung cancer is lowest [26]. One of the assumptions is that the relationship between radon concentration and risk of radon-induced lung cancer is linear and that there is no threshold, meaning that the risk of radon-induced lung cancer is lowest at 0 Bq/m^3^ [9]. Some research results indicate that the radon-induced risk of lung cancer is lowest at a minimally elevated radon concentration of around 50 Bq/m^3^ [26,27]. In our study, the lowest exposure category was defined as 0–100 Bq/m^3^, which meant that it was not possible to distinguish between the two views.

A higher risk of lung cancer due to indoor radon exposure was found in a meta-analysis of seventeen case-control studies, in which the pooled odds ratio at an indoor radon concentration of 150 Bq/m^3^ was 1.24 (95% CI: 1.11–1.38) [28]. Another meta-analysis of case-control studies showed that the pooled relative risk for lung cancer at an indoor radon concentration of 150 Bq/m^3^ was 1.14 (95% CI: 1.0–1.3) [29]. In the case-control study in eastern Germany, an odds ratio of 1.30 (95% CI: 0.88–1.93) was reported for indoor radon concentrations greater than 140 Bq/m^3^, compared with indoor radon exposure up to 50 Bq/m^3^ [30]. In the presented investigation, the indoor radon concentration on an individual level was not available. However, in our additional analysis of the relative risk of developing lung cancer by settlements (1988–2017) and municipalities (1978–1987), which were divided into two categories according to the risk of indoor radon exposure, we showed that the relative risk of developing lung cancer in the 40-year period 1978–2017 was 5% to 7% higher among the residents of the areas with a moderate or high risk of indoor radon exposure. A sub-analysis of our dataset where the moderate and high risk areas were distinguished has been performed as well. There was no significant difference in the relative risk of high or moderately exposed settlements. Our study showed that, from a public health perspective, ongoing efforts are needed in Slovenia in order to further reduce lung cancer incidence attributable to radon exposure.

We should also emphasize that our analysis is most reliable for the more recent 20-year period 1998–2017 at the settlement level due to the reliability of the information on residence at the time of diagnosis and the availability of data on the population. Another limitation of descriptive epidemiological geographical studies is using a person’s place of residence as a surrogate indicator of exposure in a study, since it might be a source of potential biases. In our research, it was not possible to account for daily mobility or migration, so using the residential address as an indicator of exposure is not an exact measure of exposure, especially if exposure occurs at the place of work or in educational institutions [31]. In addition, individuals can be exposed to environmental factors in various locations throughout the day, and some patients may have a reported residence that is different from where they actually live.

A probable risk factor is living near the uranium mine in Žirovski vrh (a settlement in the western part of Slovenia). For the period 1981–1985, an increased incidence was found in men and women in the settlements near the uranium mine [11,12]. In our analyses, we found no clusters corresponding to the uranium mine in Žirovski vrh. The most important confounding factor is smoking. It is estimated that 84% of lung cancer deaths per year in Slovenia are attributable to tobacco [32]. As an improvement to this presented study, information on smoking could be included in the analysis, because it is reported that the risk of lung cancer increases significantly with the joint effect of two risk factors: radon exposure and smoking [33,34,35,36]. In this present analysis, it is not possible to determine to what extent our results exclusively reflect the risk of indoor radon exposure and to what extent they rather reflect smoking behavior, as indoor radon exposure and smoking behavior are not geographically independent and also influence each other [33,34,35]. However, no data on smoking are available for Slovenia on a small geographical level, which imposes a severe limitation on our study. We have tried to include information on smoking by including indirect information that is available. For this reason, we have conducted an additional analysis using the SI-EDI (Slovenian version of the Socio-Economic Deprivation Index [37]) as an explanatory variable in the models to adjust the results for socioeconomic status. But the results did not provide additional insight into the topic under study, so we did not include them in the reporting of the results. This supports our earlier findings that social segregation and stratification in Slovenia is low, meaning there are no strong and extended areas where only affluent or deprived inhabitants would live [37]. The World Factbook [38] ranks Slovenia 153rd out of 157 countries, which means that the country’s income distribution is almost equal.

## 5. Conclusions

Indoor radon exposure is an important risk factor for lung cancer in Slovenia in areas with high natural radon radiation (especially in the southern and south-eastern parts of the country). In the 40-year period 1978–2017, the relative risk for lung cancer was 5–7% higher in areas with a moderate or high risk of indoor radon exposure. This resulted in 60 lung cancer cases per year on average attributable to the risk of indoor radon exposure in the residential environment, which corresponds to 4.9% of lung cancer cases (according to the analysis by settlement).

It is known that the geology in some parts of Slovenia is the cause for the increased risk of indoor radon exposure. In recent decades, many measures have been implemented in building regulations and in efforts to educate people about the hazards and about preventive behavior. Nevertheless, our study showed that, from a public health perspective, ongoing efforts are needed in Slovenia in order to further reduce lung cancer incidence attributable to radon exposure.

## Figures and Tables

**Table 1:** Incidence of lung cancer, average annual incidence of lung cancer, average annual population size, and the percentage of the population living in settlements with a moderate or high risk of indoor radon exposure over four consecutive 10-year periods.

10-Year Period	Incidence	Average Annual Incidence	Average Annual Population	% of Population Living in Areas with a Moderate or High Risk of Indoor Radon Exposure
1978–1987	7201	720	1,891,864	15.1% *
1988–1997	9426	943	1,965,986	15.1%
1998–2007	11,324	1132	1,978,897	15.5%
2008–2017	13,304	1330	2,052,376	15.9%
1978–2017	41,255	1031	1,972,281	15.4%

* Calculated for municipalities since data on settlement level is not available for period 1978–1987.

**Table 2:** Incidence of lung cancer for consecutive 10-year periods from 1978 to 2017 and the population attributable fraction (PAF) to radon exposure according to the analysis by settlement and municipalities.

		Settlements		Municipalities	
	Incidence	PAF	Attributable Incidence	PAF	Attributable Incidence
2008−2017	13,304	4.3%	572	6.5%	865
1998−2007	11,324	5.6%	634	6.2%	702
1988−1997	9426	1.2%	113	2.8%	264
1978−1987	7201	/ *	/ *	6.2%	447

* Data not available on settlement level.

**Table 3:** Standardized incidence ratio (SIR) with a 95% confidence interval for lung cancer by sex and by areas for indoor radon exposure in consecutive 10-year periods from 1978 to 2017 in Slovenia.

		Males	Females	Males and Females
2008–2017				
Risk of radon exposure	low	0.99 [0.97–1.01]	1.00 [0.97–1.03]	0.99 [0.97–1.01]
	moderate or high	**1.05 [1.00–1.13]**	1.01 [0.94–1.09]	**1.05 [1.00–1.09]**
1998–2007				
Risk of radon exposure	low	0.98 [0.96–1.01]	1.00 [0.96–1.04]	0.99 [0.97–1.01]
	moderate or high	**1.08 [1.03–1.14]**	0.98 [0.89–1.09]	**1.07 [1.02–1.12]**
1988–1997				
Risk of radon exposure	low	0.98 [0.96–1.01]	1.01 [0.95–1.06]	0.99 [0.97–1.01]
	moderate or high	**1.09 [1.03–1.15]**	0.97 [0.85–1.10]	**1.07 [1.02–1.12]**
1978–1987				
Risk of radon exposure	low	0.98 [0.95–1.01]	1.00 [0.94–1.07]	0.98 [0.96–1.01]
	moderate or high	**1.07 [1.01–1.13]**	0.99 [0.86–1.12]	**1.05 [1.00–1.11]**

The results in bold are statistically significant-the lower limit of the 95% confidence interval was above one.

## Data Availability

Detailed results are available from the corresponding author upon reasonable request.
